# Is Helicobacter Pylori a Reason for Unexplained Iron Deficiency Anemia: A Systematic Review

**DOI:** 10.7759/cureus.29112

**Published:** 2022-09-13

**Authors:** Jiya Mulayamkuzhiyil Saju, Naishal Mandal, Nang I Kham, Rabia Shahid, Shaili S Naik, Shivana Ramphall, Swarnima Rijal, Vishakh Prakash, Heba Ekladios, Pousette Hamid

**Affiliations:** 1 Internal Medicine, California Institute of Behavioral Neurosciences & Psychology, Fairfield, USA; 2 Psychiatry, California Institute of Behavioral Neurosciences & Psychology, Fairfield, USA; 3 Neurology, California Institute of Behavioral Neurosciences & Psychology, Fairfield, USA

**Keywords:** anti h pylori therapy, serum ferritin, unexplained anemia, iron deficiency anemia, h pylori

## Abstract

Iron deficiency anemia (IDA) is a worldwide public health problem affecting millions, with developing nations accruing a significant disease burden. *Helicobacter pylori* (*H. pylori*) has been proposed in many studies as a causative factor for unexplained iron deficiency anemia. In this systematic review, we searched PubMed, Google Scholar, and ScienceDirect to come up with five cross-sectional studies and five Randomized Controlled Trials (RCTs), which evaluated the association between *H. pylori* and unexplained iron deficiency anemia and the response of IDA to anti-*H. pylori* therapy. *H. pylori* eradication therapy included triple therapy (proton pump inhibitor, clarithromycin, amoxicillin) or quadruple therapy (proton pump inhibitor, bismuth, metronidazole, tetracycline) for 10-14 days. Quadruple therapy was used if there is a penicillin allergy or a local antibiotic resistance level of more than 15% to clarithromycin. The cross-sectional studies concluded that *H. pylori* infection was associated with low serum ferritin levels. The RCTs confirmed that *H. pylori* are associated with iron deficiency anemia by demonstrating improvement in markers of iron status (ferritin, hemoglobin, Mean Corpuscular Volume (MCV), serum transferrin receptor levels) with *H. pylori* eradication therapy. In a nutshell, this systematic review concludes that *H. pylori* testing and treatment must be considered as a differential diagnosis of unexplained IDA in all age groups and serves as a benchmark for more randomized clinical trials to prove causation.

## Introduction and background

Anemia is a global public health problem, mostly in developing nations. World Health Organization (WHO) estimates that over 700 million people worldwide have iron deficiency anemia (IDA) [1]. Poor iron intake, gastrointestinal parasitic infections, low bioavailability of dietary iron, defective iron absorption, and excessive iron loss are the major factors contributing to iron deficiency anemia [2-3]. Iron deficiency in children is associated with deficits in immune, cognitive, and motor function [4]. Unexplained or refractory iron deficiency anemia accounts for about 15% of all IDA [5].

*Helicobacter pylori (H. pylori)* has been postulated as an etiology for unexplained iron deficiency anemia. The prevalence rate of *H. pylori *infection is 50% in adults in developed countries and 60%-90% in developing countries [6]. *H. pylori*-induced chronic gastritis depletes gastric acid secretion and gastric ascorbic acid levels, which are necessary for iron absorption [7-8]. Patients with *H. pylori* infection show high intragastric pH leading to defective iron absorption and consequent IDA [9]. Other causes of IDA are the sequestration of iron by *H. pylori *and the loss of iron through the gastrointestinal tract because of inflammatory mucosal injury [8]. Children in developing nations are more likely to develop iron deficiency anemia from *H. pylori* infection as they may have diminished iron stores due to dietary deficiency [10]. 

Many studies evaluating the association between *H. pylori *infection and IDA are cross-sectional. There are also some randomized controlled trials assessing the response of markers of iron status to *H. pylori* eradication therapy in infected individuals with or without IDA. Some of these studies have been performed on the Alaskan native population because 20%-50% of them continue to have depleted iron stores despite adequate dietary iron and Vitamin C intake [11]. Anti*-H. pylori *therapy includes standard triple therapy in which a proton pump inhibitor (PPI), amoxicillin, and clarithromycin are given for 10-14 days to eradicate the infection. In case of penicillin allergy, modified triple therapy with replacement of amoxicillin with metronidazole can be given, while PPI and clarithromycin can continue. The treatment regimen for *H. pylori* depends on local antibiotic susceptibility. In areas of more than 15% macrolide (clarithromycin) resistance, quadruple therapy is initiated, which includes treatment with PPI, bismuth, metronidazole, and tetracycline for 10-14 days. The quadruple regimen can also be used if there is treatment failure on triple therapy. In this systematic review involving global studies, the role of *H. pylori *as a cause of unexplained IDA and the usefulness of *H. pylori* eradication therapy in improving IDA or its parameters are explored.

## Review

Method

Search strategy

This study was undertaken per PRISMA (Preferred Reporting Items for Systematic Reviews and Meta-analysis) guidelines [12]. Databases included were PubMed, Google Scholar, and ScienceDirect. We searched for all Publications on H. Pylori and iron deficiency anemia between 1995 to 2022. A database search was performed on PubMed using the following regular keywords: *Helicobacter Pylori*, Unexplained iron deficiency anemia, Refractory iron deficiency anemia, Microcytic anemia, Atrophic gastritis. The following Medical Subject Headings (MeSH) keywords were used to conduct a literature search in PubMed: 

*Helicobacter pylori* (( "*Helicobacter pylori*/etiology"[Majr] OR "*Helicobacter pylori*/metabolism"[Majr] OR "*Helicobacter pylori*/pathogenicity"[Majr] )) OR ( "*Helicobacter pylori*/etiology"[Mesh:NoExp] OR "*Helicobacter pylori*/metabolism"[Mesh:NoExp] OR "*Helicobacter pylori*/pathogenicity"[Mesh:NoExp] ) AND Unexplained iron deficiency anemia (( "Anemia, Iron-Deficiency/diagnosis"[Majr] OR "Anemia, Iron-Deficiency/drug therapy"[Majr] OR "Anemia, Iron-Deficiency/etiology"[Majr] OR "Anemia, Iron-Deficiency/microbiology"[Majr] OR "Anemia, Iron-Deficiency/pathology"[Majr] OR "Anemia, Iron-Deficiency/physiopathology"[Majr] OR "Anemia, Iron-Deficiency/prevention and control"[Majr] OR "Anemia, Iron-Deficiency/therapy"[Majr] )) OR ( "Anemia, Iron-Deficiency/diagnosis"[Mesh:NoExp] OR "Anemia, Iron-Deficiency/drug therapy"[Mesh:NoExp] OR "Anemia, Iron-Deficiency/etiology"[Mesh:NoExp] OR "Anemia, Iron-Deficiency/microbiology"[Mesh:NoExp] OR "Anemia, Iron-Deficiency/pathology"[Mesh:NoExp] OR "Anemia, Iron-Deficiency/physiopathology"[Mesh:NoExp] OR "Anemia, Iron-Deficiency/prevention and control"[Mesh:NoExp] OR "Anemia, Iron-Deficiency/therapy"[Mesh:NoExp] )

The search included free full-text articles- observational studies, systematic reviews, or randomized clinical trials conducted on human subjects and published in English from 1995 to 2022. We also used Google Scholar and ScienceDirect to find relevant articles on H. Pylori and iron deficiency anemia. We also used reference lists of original articles and the 'Related Articles' section of PubMed to find relevant articles.

Study selection

Two reviewers independently screened the titles and abstracts of all identified studies for eligibility. The following inclusion criteria were applied: (1) cross-sectional studies, case-control studies, systematic review articles, randomized controlled trials, and meta-analyses (2) all age groups regardless of gender (3) any geographic regions of the world.

Case reports/series, books, letters to the editor, conference abstracts, author's replies, and editorials were excluded. Those articles which required permission for access or payment requirement were also excluded. Articles related to iron deficiency anemia in the pregnant population and anemia of chronic disease were excluded.

Data extraction

Data were extracted independently by two reviewers who initially screened the titles and abstracts for eligibility. A full-text review of the eligible studies was done by two authors independently according to eligibility criteria. The following data were extracted: Study, study design, study population, age of participants, number of participants, the prevalence of *H. pylori* infection, classification of Iron Deficiency (ID)/ Anemia/Iron Deficiency Anemia (IDA), and *H. pylori* detection method.

For cross-sectional studies, we extracted the data on the *H. pylori* infection status of study participants based on Immunoglobulin G (IgG) antibody levels/urea breath test/stool antigen test/endoscopy, the prevalence of *H. pylori* infection in percentage, the measures of association between *H. pylori* infection and iron deficiency anemia using Odds Ratio (OR) or relative risk (RR). For randomized clinical trials, data were collected regarding the number of participants in the intervention and control group, the treatment received by each group, and the baseline and follow-up levels of markers of iron status, including serum ferritin (SF), hemoglobin (Hb), Mean Corpuscular Volume (MCV) and serum transferrin receptor (sTfR) levels. Any significant association between *H. pylori* infection and the markers of iron status, which demonstrated a p-value of less than 0.05, was considered to interpret the results of this systematic review.

Methodological quality assessment

The risk of bias in studies included in this systematic review was assessed independently by two reviewers, and disagreements were resolved by consensus. Quality assessment of cross-sectional studies was done using the Adapted Newcastle Ottawa Quality Assessment Scale developed by Herzog et al. [13]. The scale included three main categories: selection, comparability, and outcome. A maximum of one point is given for each numbered item in the selection and exposure category, and a maximum of two points is given for comparability. This contributes to a maximum score of four, two, and two, respectively, in the selection, comparability, and outcome categories of the Adapted Newcastle Ottawa Quality Assessment Scale. The Cochrane Collaboration's tool was used for the quality assessment of randomized clinical trials included in this review [14]. The bias was assessed as high, low, or unclear for seven individual elements spread over five domains (selection, performance, attrition, reporting, and others).

Results

Literature search

**Figure 1:**
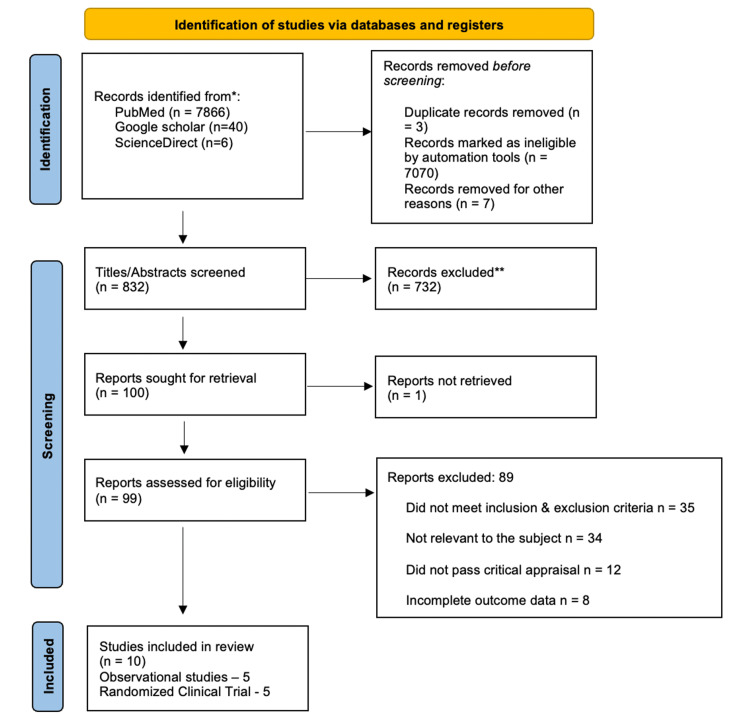
PRISMA 2020 flow diagram PRISMA = Preferred Reporting Items for Systematic Reviews and Meta-Analyses

A flow diagram of study identification and subsequent inclusion is shown in Figure 1. A total of 7912 citations were identified by the initial search, in which 10 studies were selected for this systematic review after the screening, eligibility criteria, and quality assessment. A total of 7144 patients were included in this study.

Characteristics of the included studies

The characteristics of the included studies are shown in Table 1. These studies spanned from 1998 to 2021 and were done in different parts of the world. The number of participants varied from a minimum of 86 to a maximum participant number of 2794. Both children and adults were included in our study. Five studies evaluated participants' responses to H. pylori eradication therapy concerning iron deficiency anemia. Four studies among them were randomized controlled trials [6,9,15-16], and one was an uncontrolled trial [17]. Five out of ten studies chosen for systematic review were cross-sectional studies [4,7-8,10,18]. Among the studies, three were conducted in Alaska [8,15,18], three were conducted in Asia [6-7,9], and others in Europe [10], the United States [16], and Egypt [17]. The study by Queiroz et al. [4] was an international multicenter trial conducted in South American countries (Chile, Brazil) and Europe (UK).

**Table 1:** Study characteristics ID: Iron deficiency (determined by serum ferritin levels in most studies); Anemia: Determined by hemoglobin levels in most studies; IDA: Iron deficiency anemia (determined by all or some of these parameters in most studies: Hemoglobin, transferrin saturation, MCV, ferritin);  EIA: Enzyme Immunoassay;  UBT: Urea breath test; RCT: Randomized controlled trial; MCV: Mean corpuscular volume; MCH: Mean corpuscular hemoglobin;  ELISA: Enzyme-linked immunosorbent assay;  IgG: Immunoglobin G;  sTfR: Serum transferrin receptor; *H. pylori: Helicobacter pylori;  *S. ferritin: Serum ferritin;  Hb: Hemoglobin

Study	Study design	Study Location	Number of participants	Age of participants	Prevalence of *H. pylori* infection %	Definition of markers of iron status	*H. pylori* infection detection method
DiGirolamo et al. [8]	Cross-sectional study	Alaska	86	1-5 years	41% IgG positive, 86% UBT positive, 80% Stool antigen test positive	ID - Ferritin<10 ng/mL; IDA = Hb <11 g/dL for <2 years, <11.1 g/dL for 2-5 years, <11.5 g/dL for 5 year	*H. pylori* IgG, UBT, Stool antigen EIA
Lee et al. [7]	Retrospective cohort	Korea	281	22-65 years	66.50%	ID - Ferritin < 30 ng/mL; Anemia Hb <13 g/dL for men, <12 g/dL for women; IDA – Anemia + Ferritin <10 ng/mL, transferrin saturation <16%	CLO test (rapid urease test )conducted during endoscopy
Milman et al. [10]	Cross-sectional study (Population-based survey)	Denmark	2794	30-60 years	25.60%	ID - Ferritin < 15 mg/L; Anemia Hb <13 g/dL for men, <12 g/dL for women; IDA - Ferritin <12 mg/L	*H. pylori* IgG by ELISA
Parkinson et al. [18]	Cross-sectional study (Population-based serosurvey)	Alaska	2080	<5 to 50+ age groups	75%	<12 ng/mL for low serum ferritin	*H. pylori* IgG by ELISA
Queiroz et al. [4]	International multicentered study - Cross-sectional study	Brazil, Chile, United Kingdom	311	10.7+-3.2 mean age, 3-16 years	27.70%	IDA – Hb <110 g/L (3-5 years), <115 g/L (6-11 years), <120 g/L (12-16 years), S. ferritin <12 mg/L (3-5 years), <15 mg/L (6-16 years)	*H. pylori* by endoscopy and culture/biopsy urease test/Giemsa staining of histology section
Cardenas et al. [16]	RCT - Double-blind	Texas	110	3-10 years	All children selected were *H. pylori* infected but not iron deficient.		*H. pylori* IgG by urine test f/b UBT, Anti Cag A antibody by EIA at 6-month f/u
Fagan et al. [15]	RCT - Open-label	Alaska	219	7-11 years	All children were iron deficient and *H. pylori*-positive	ID <10 mg/L of ferritin; Anemia- Hb <115 g/L; IDA – iron <25mg/dL, TIBC >450 mg/dL, iron saturation<15%	*H. pylori* by UBT with hydrolysis rate >10 mg/min
Xia et al. [6]	RCT - Double-blind	Suihua, China	1037	12–18-year-old females	31.2% both stool antigen test and IgG antibody test positive	ID- S.ferritin <12 mg/L; Anemia - Hb<120 g/L; IDA – Hb <120 g/L, S.ferritin <12 mg/L or Hb <120 g/L, S.ferritin >12 mg/L, sTfR >24.5 mmol/L	Current *H. pylori* infection: Stool Antigen positivity + *H. pylori* IgG antibody positivity
Chen et al. [9]	RCT	Wuhan, China	86	18-76, median age 53	All 86 were having IDA + *H. pylori* positive chronic gastritis	IDA - Hb <120 g/L in men & <110 g/L in women, S. ferritin < 12 mg/L, MCH < 27 pg, MCV < 80 fL	UBT > 3.33 Bq C14 content considered positive, gastritis diagnosed by endoscopy
Kurekci et al. [17]	Uncontrolled clinical trial	Egypt	140	6-16 years	All 140 were *H. pylori* positive	ID - Ferritin <10 ng/mL; IDA - Ferritin <10 ng/mL, Hb & MCV below the lower limit of age matched values; Control - Normal Hb + S.ferritin>10 ng/mL	*H. pylori* eradication is considered if both stool Antigen test & UBT are negative

Risk of bias assessment

Table 2 demonstrates the risk of bias assessment for randomized controlled trials done by the Cochrane Collaboration's tool [14]. Among the RCT, three studies depicted a low risk of bias [6,15-16], and two studies [9,17] had an unclear risk of bias with low risk in at least two domains. 

**Table 2:** Cochrane Collaboration's tool for risk of bias assessment of Randomized Controlled Trial Risk of bias: L: Low, H: High, U: Unclear

	Random Sequence Generation - Selection bias	Allocation of concealment - Selection bias	Blinding of both participants and evaluators - Performance bias	Blinding of assessment during outcome collection - Detection bias	Incomplete outcome data - Attrition bias	Selective reporting - Reporting bias	Other bias / Comments
Cardenas et al. (2011) [16]	L	L	L	L	L	L	L
Fagan et al. (2009) [15]	L	L	H	H	L	L	L
Xia et al. (2011) [6]	L	L	L	L	L	L	L
Kurekci et al. (2005) [17]	H	U	U	U	U	L	L
Chen et al. (2007) [9]	L	U	U	U	U	U	L

Table 3 demonstrates the risk of bias assessment for cross-sectional studies using the Adapted Newcastle Ottawa scale [13]. Three studies were classified as good, with a total score of 7-8 [4,8,10]. Two studies were satisfactory, scoring 5-6 [7,18].

**Table 3:** Adapted Newcastle Ottawa Scale for risk of bias assessment of Cross-sectional study

	Selection (maximum 4)				Comparability (maximum 2)	Outcome (maximum 2)		Total Score
	Representativeness of the sample	Sample size	Non-respondents	Ascertainment of the exposure (absence or exclusion)	The subjects in different outcome groups are comparable, based on the study design or analysis. Confounding factors are controlled	Assessment of the outcome	Statistical test	
DiGirolamo et al. [8]	1	1	1	1	1	1	1	7
Milman et al. [10]	1	1	1	1	2	1	1	8
Parkinson et al. [18]	1	1	0	1	1	1	1	6
Queiroz et al. [4]	1	1	0	1	2	1	1	7
Lee et al. [7]	1	1	0	1	0	1	1	5

Observational studies evaluating the association between H. pylori infection and iron deficiency anemia

Five observational studies/cross-sectional studies evaluated the association between *H. pylori *infection and iron deficiency anemia.

In the study by DiGirolamo et al., which was conducted on children of Southwest Alaska, the age of participants ranged from 1 to 5 years [8]. The mean age of study participants was 43.7 months, with a standard deviation of 16.8 months. A total of 86 participants were included in this population-based survey. 41% (34 of 83) of children tested positive for *H. pylori* specific IgG, 86% (74 of 86) tested positive on the urea breath test (UBT), and 80% (41 of 51) tested positive on the stool antigen test. It was found that *H. pylori* seropositivity increased with age (c2 - 4.6, p < 0.05). This study's overall prevalence of iron deficiency and iron-deficiency anemia (IDA) were 39% and 15%, respectively. Di Girolamo et al. found a positive association between the presence of *H. pylori* seropositivity (IgG antibody positivity) and IDA (Crude Odds Ratio 4.91, CI 1.23 - 19.61) [8]. The adjusted Odds ratio for this association was 6.99, CI 1.46 - 33.58, after controlling for age, gender, recent antibiotic use, and C-Reactive Protein (CRP). Contrary to this, children with positive UBT or stool antigen tests exhibited a lower prevalence of iron deficiency or IDA than children with negative UBT or stool antigen tests.

Lee et al. conducted a retrospective study on 281 adults aged 22 to 65 who attended a health check-up in a hospital in Korea from 2016 to 2017 [7]. The study used the CLO test or rapid urease test to diagnose *H. pylori* infection, which was done on endoscopic biopsy specimens taken from the antrum or body of the stomach. The mean age of study participants was 36.1 years, and 62.6% (176 of 281) were male. 66.5% (187 of 281) were positive for *H. pylori* and majority of them were female subjects (74.3% Vs 61.9%, p = 0.034). The study found that the mean levels of hemoglobin (14.1 ± 1.7 g/dL vs. 14.6 ± 1.4 g/dL, p = 0.019) and ferritin (121.7 ± 106.9 ng/mL vs. 151.8 ± 107.8 ng/mL, p = 0.027) were significantly lower in the *H. pylori*-positive group compared to *H. pylori*-negative group. In this study, iron deficiency (ferritin < 30 ng/mL) was more common in patients with *H. pylori* infection (p = 0.002). In contrast, anemia (defined as Hb < 13 in males and < 12 in females, p = 0.225) and iron deficiency anemia (defined as anemia, ferritin < 10 ng/mL, transferrin saturation < 16%, p = 0.173) did not correlate significantly with *H. pylori* infection status. In univariate analysis, female sex (Odds ratio 192.500; 95% CI 25.986 - 1425.981, p<0.001) and *H. pylori* infection (Odds ratio 3.171, 95% CI 1.479 - 6.796, p = 0.003) correlated with iron deficiency.

Milman et al. conducted a cross-sectional study on 2794 Danish populations aged between 30 to 60 years [10]. This questionnaire-based survey was conducted between 1982-1984. It was adjusted for age, gender, menopausal status, socio-economic status, blood donation activity, hormonal contraception, non-steroidal antiinflammatory drugs (NSAIDs), peptic ulcer disease history, and cumulated weekly number of alcoholic drinks. As per this study, the prevalence of *H. pylori* infection in the population was 25.6%, and the seroprevalence increased with age in both men and women. The study found that serum ferritin was lower (<30 mg/L) in *H. pylori* IgG positive people compared to *H. pylori* IgG negative participants, and this difference persisted in multivariate logistic regression analysis (Odds ratio 1.4, CI 1.1 to 1.8). As per this study, *H. pylori* IgG positive men (114 mg/L vs. 120 mg/L, p = 0.01) and postmenopausal women (63 vs. 77 mg/L, p = 0.02) had lower median serum ferritin levels compared to respective IgG negative category. This difference was not significant in pre-menopausal women (p = 0.13). The study also found that *H. pylori* seropositive postmenopausal women were more likely to have iron deficiency (serum ferritin < 15 mg/L) compared to seronegative postmenopausal women (p = 0.03).

Another population-based serosurvey by Parkinson et al. evaluated the association between *H. pylori* infection and serum ferritin in 2080 Alaskan natives [18]. The study was conducted between 1980-1986 in stored serum samples of participants stratified into <5, 5-9, 10 to 14, 15 to 19, 20 to 29, 30 to 39, 40 to 49, and 50+ age groups. Overall IgG positivity rate in the population as per this study was 74.8% (1556 of 2080) when the indeterminate samples of 170 were added to the truly positive 1386 samples. This study demonstrated that *H. pylori* seropositivity increased with age (z - 15.05, p <0.001), which was 32% (84 of 260) in the 0-4-year age group and increased to 77.7% (202 of 260) in the 10-14 age group. Low serum ferritin of <12 ng/mL was found in 19.6% of males and 35.8% of females. Among those with the low serum ferritin, 73.5% of males and 73.6% of females were seropositive for *H. pylori*. The relative risk of having low serum ferritin levels (<12 ng/mL) in *H. pylori* seropositive individuals compared to IgG negative individuals was 1.13 (c2 - 6.24[MH test], p = 0.013), after adjusting for age and gender. This association was even more significant for persons aged less than 20 years (RR 1.15, c2 - 10.0 [MH test]; p< 0.002).

An international multicentered study by Queiroz et al. evaluated the effects of *H. pylori* infection on iron deficiency in a cohort of 311 children aged 3-16 years in Santiago in Chile, Belo Horizonte in Brazil, and London in the UK [4]. The study confirmed *H. pylori* positivity if endoscopic biopsy followed by culture was positive or if endoscopic biopsy urease test and stained histology sections were positive. *H. pylori* infection status was confirmed negative if all three tests were negative. The overall prevalence of *H. pylori* infection was 27.7% (86 of 311). The prevalence was significantly higher in Santiago and Belo Horizonte than in London (p < 0.001 for both). This study did not find a significant correlation between *H. pylori* infection and low hemoglobin and ferritin in a combined analysis of all three countries by linear regression. In the combined population analysis of Chile and Brazil, due to similar socio-economic development, *H. pylori* infection and female gender were significant predictors of low serum ferritin (p = 0.01) and hemoglobin (p = o.o4) in multiple linear regression models. In this study, a negative correlation was observed between MCV (r = -0.26, p = 0.01) and Mean Corpuscular Hemoglobin (MCH) (r = -0.27, p = 0.01) values and degree of chronic inflammation in the antrum in *H. pylori*-positive children. Similar negative correlation was observed between MCH and the degree of chronic (r = -0.29, p = 0.008) and active inflammation (r = -0.27, p = 0.002) in the corpus of stomach.

Trials evaluating response to H. pylori eradication therapy influencing iron-related parameters

Cardenas et al. conducted a double-blind, randomized controlled trial in El Paso County, Texas, between 2006 and 2008 in 110 *H. pylori*-infected children without iron deficiency and between 3 to 10 years [16]. The intervention consisted of quadruple sequential treatment of lansoprazole and amoxicillin for the first five days, followed by lansoprazole + clarithromycin + tinidazole for the next five days. Ferrous sulfate supplementation consisted of 3 mg/kg/day of elemental iron for six weeks. Four treatment arms were defined: quadruple eradication + iron supplementation, quadruple eradication only, iron supplementation only, and placebo. In the intent to treat (ITT) analysis, subjects received at least one dose of *H. pylori* eradication treatment. As per ITT analysis, only 44% of children receiving *H. pylori* eradication alone or combined with iron cleared their infection. Per protocol (PP) analysis accounted for losses to follow-up and included 90 children. In both ITT (n = 110) and PP (n = 90) analysis, there were no significant differences across study arms on means and mean differences of hemoglobin, serum ferritin, or transferrin saturation from baseline levels at six months follow-up. However, the children who were eradicated from *H. pylori* at 45 days showed a statistically significant larger increase in mean ferritin levels (7.7 ng/mL, p <0.05) than children who remained infected (1.9 ng/mL). The study also showed that eradicating infection by CagA (cytotoxin associated gene product A) negative strain showed a larger increase in ferritin than CagA positive strain.

Fagan et al. conducted an open-label randomized controlled trial on 219 children aged 7-11 years in the Western Alaska region who were both iron deficient and *H. pylori*-positive [15]. These children were randomized to receive either iron sulfate alone (113 children in the control group) or iron sulfate plus *H. pylori* treatment (106 children in the intervention group). The prevalence of iron deficiency (ARR 0.62, CI 0.38-1.01) and iron deficiency anemia (ARR 0.22, CI (0.03-1.50) in *H. pylori*-negative children at 40 months was lower compared to *H. pylori*-positive children, but the result did not attain statistical significance. However, children who became *H. pylori*-negative at 40 months demonstrated statistically significant improvement in median ferritin level (29.2 vs. 20.2, p = 0.005) and median change in ferritin from baseline (14.8 vs. 6.6, p = 0.005) compared to *H. pylori*-positive group. Median hemoglobin level (p = 0.268) and medium change in hemoglobin level (p = 0.381) at 40 months did not demonstrate a statistically significant difference in the *H. pylori*-negative group compared to the *H. pylori*-positive group.

Xia et al. conducted a double-blind, randomized controlled trial in 1037 adolescent girls aged 12-18 in northeast China to evaluate the relationship between *H. pylori* infection and iron deficiency anemia [6]. According to this study, the prevalence of iron deficiency, anemia, IDA, and *H. pylori* infection in the population were 40.4%, 19.5%, 17.1%, and 31.2%, respectively. 46.9% (83 of 177) in the IDA group were *H. pylori*-positive compared to 28.1% (235 of 835) in the non-anemic group, and this difference was statistically significant (p <0.01). In multiple logistic regression analysis, a large volume of menstruation (r 0·239, OR = 1·270, 95 % CI 1·059, 1·523) and *H. pylori* infection (r 0·347, OR = 1·360, 95 % CI 1·212, 1·638) were found as risk factors for anemia. Eighty adolescent girls with IDA and *H. pylori* infection were randomized into the intervention group (oral iron + triple therapy, n = 32) and control group (oral iron only, n = 42) after exclusion criteria. It was found that the mean values of Hb and SF increased and sTfR decreased after treatment compared with those before treatment in the intervention group (p <0.01). This difference was maintained in the *H. pylori*-negative group (n = 31, p <0.01) after treatment compared to before therapy after re-division based on *H. pylori* eradication status following the intervention trial.

Chen et al. conducted a randomized controlled trial in 86 adult patients with iron deficiency anemia and *H. pylori*-associated chronic gastritis. They evaluated the effect of *H. pylori* eradication on the response to oral iron therapy by dividing them into two equal-sized groups of 43 each [9]. Group A cases received both oral iron treatment and *H. pylori* eradication therapy, while Group B only received oral iron treatment. The mean Hb, MCH and MCV were recorded at days 0,7,14,21,28,35,42,49 and 56 and the serum iron and ferritin were recorded at days 0,14,28,42 and 56. The mean Hemoglobin (Hb) in group A returned to normal (>120 g/L in men & >110 g/L in women) on day 56, and the levels were higher than those in group B (p<0.05). The MCV (>80 fL) and MCH (>27 pg) in group A became normal on day 21, and the difference was significant when compared to group B on day 21 (p< 0.05). The serum ferritin (p < 0.01) and serum iron levels (p <0.05) were higher in group A compared to group B on day 56.

Kurekci et al. studied 140 children aged 6-16 years to know whether *H. pylori* eradication without iron treatment would lead to the resolution of ID and IDA [17]. The study subjects were divided into three groups, namely, Group I (IDA, n = 18), Group II (ID, n = 36), and Group III (Control, n = 86). They were treated with two-week *H. pylori* eradication therapy. Subjects were concluded to be *H. pylori* eradicated if both stool antigen test and urea breath test were negative four weeks after completion of triple therapy. The study concluded that Hb (p < 0.002) and MCV (p < 0.003) values increased significantly after treatment in children with IDA. Serum ferritin levels increased significantly post-treatment in all groups, i.e., ID, IDA, and control groups (p<0.001).

Discussion

This systematic review found an association between *H. pylori* infection and iron deficiency anemia. Two forms of iron are absorbed in the duodenum and jejunum part of the gastrointestinal tract: heme iron and non-heme iron. Heme iron is mainly present in meat and is easily absorbed [7]. Non-heme iron is primarily present in vegetables and grains and requires conversion from the oxidized ferric form to the ferrous form to get absorbed [10]. The reduction of ferric to the ferrous form of iron is facilitated by acidic gastric pH and ascorbic acid. *H. pylori* increase gastric pH by causing atrophic gastritis, uses iron for its growth and acts as a causative factor for iron deficiency anemia. The outer membrane proteins in *H. pylori* bind ferritin and lactoferrin with high affinity [19-20]. *H. pylori* can also alter iron metabolism by iron sequestration [21]. Occult gastrointestinal bleeding caused by *H. pylori*-induced peptic ulcer disease is a less common cause of iron deficiency anemia than the causes mentioned above. It has been proposed by the Maastricht V/Florence Consensus Report that patients with unexplained iron deficiency anemia must be tested and treated for *H. pylori* infection. However, the grade of recommendation is weak [22].

*Association between H. pylori infection and iron deficiency anemia* 

Among the cross-sectional studies included in this systematic review, while Di Girolamo et al. found a correlation between *H. pylori* IgG seropositivity and iron deficiency anemia, Lee et al. could not find a significant association between *H. pylori* infection status and iron deficiency anemia [7-8]. But Lee et al. commented that the mean hemoglobin and ferritin levels were lower in the *H. pylori*-positive group than in the negative group [7]. Milman et al. concluded that serum ferritin was lower in *H. pylori* IgG-positive people (OR-1.4, CI 1.1 - 1.8), and this was confirmed by Parkinson et al. (RR - 1.13 (c2 - 6.24 [Mantel-Haenszel test](MH test), p = 0.013) and these studies were adequately powered with a sample size of more than 2000 study subjects [10,18]. Queiroz et al., in their multicenter study, found an association between *H. pylori* infection and low serum ferritin and hemoglobin in a combined analysis of the population of Chile and Brazil, which were nations with similar socio-economic development [4].

All the randomized clinical trials included in this systematic review concluded that *H. pylori* eradication therapy increased serum ferritin levels from baseline. While Cardenas et al. [16] and Fagan et al. [15] did not report an increase in mean hemoglobin levels in the *H. pylori* eradicated patients, other studies [6,9,17] demonstrated that there was an increase in hemoglobin levels in subjects who became *H. pylori*-negative upon treatment.

Three cross-sectional studies included in this review reported that the seroprevalence of *H. pylori* in terms of IgG positivity increased with age [8,10,18]. This was identical to the results obtained by Lim et al. in their nationwide multicenter study conducted in Korea, which reported that the seroprevalence of *H. pylori* increased linearly from 20 (26.4%) to 59 (61.4%) years of age [23].

All the cross-sectional studies included in this review and the RCT by Xia et al. commented upon the prevalence of *H. pylori* infection in the population they studied. The prevalence of *H. pylori* infection ranged from 25.6%, as reported by Milman et al., to 75%, as written by Parkinson et al. [10,18]. The high prevalence of *H. pylori* infection in Alaskan natives, as reported by Parkinson et al., was also studied by Miernyk et al., who reported a prevalence of 69% by urea breath test (UBT) and 68% by anti-*H.-pylori* IgG [24]. A similar trend is seen in developing nations and may be attributed to the socio-economic status, crowded living places, and the unavailability of piped water supply [24].

H. pylori infection and its influence on serum ferritin levels

All the studies included in this review concluded a correlation between *H. pylori* infection and low serum ferritin levels. In a large-scale survey of 7462 participants, *H. pylori* infection was associated with a 13.9% decrease in serum ferritin levels and with the prevalence of IDA (OR 2.6, CI 1.5 - 4.6) [25]. A cross-sectional study conducted by Berg et al. in Germany also reported a 17% decrease in serum ferritin (CI 9.8 - 23.6) in the *H. pylori*-infected group in multiple regression analysis in a sample size of 1806 individuals. Berg et al. also found a similar decrease in serum ferritin levels in infection with both Cag-A positive and negative strains [19]. This is contrary to the findings of Cardenas et al., who found a significant increase in ferritin levels with the eradication of infection in the Cag-A negative strain [16].

The increase in serum ferritin level after *H. pylori* eradication therapy, as depicted by the clinical trials included in this review, is endorsed by the studies of Choe et al. [26]. Metanalysis by Qu et al. suggested that IDA is associated with *H. pylori* (pooled OR 2.22, 95% CI 1.52 -3.24 (p < 0.0001), and the eradication of *H. pylori* improves IDA [27]. These conclusions were contradicted by the studies of Gessner et al., who documented that the resolution of *H. pylori* infection did not improve iron deficiency or anemia up to 14 months after treatment initiation [28]. A possible explanation is that atrophic gastritis caused by *H. pylori* infection leading to iron deficiency anemia may take more than 14 months for resolution. Tseng et al. did not find significant improvement in IDA after *H. pylori* treatment when followed up for up to two years [29]. A retrospective cross-sectional study in older adults did not demonstrate an association between iron deficiency anemia and *H. pylori* infection [30].

Since all of the trials show an improvement in ferritin levels with therapy, our analysis stresses that ferritin is the first marker of iron status to improve with the removal of *H. pylori* and iron supplementation. The most effective test for iron deficiency is serum ferritin, which indicates the body's mobilizable iron reserves [10]. Our study emphasizes the relevance of implementing a national guideline for treating refractory iron deficiency anemia with *H. pylori* eradication therapy. This review reflects on the necessity to address the burden of unexplained anemia in the community by addressing the impact of the highly prevalent *H. pylori* infection.

Limitations

One of the limitations of our study was the cross-sectional design of five of the included studies. Also, we could not demonstrate an improvement in iron deficiency anemia in some of the clinical trials included in the analysis. However, we were able to emphasize an improvement in serum ferritin and hemoglobin levels with anti-*H. pylori* therapy. Also, we could not tailor our study to specific populations with a high incidence of iron deficiency anemia, namely pregnant and pre-menopausal women. Also, the increase in serum ferritin seen in anemia of chronic disease could not be accounted for in our study. However, this systematic review includes large-scale cross-sectional and randomized trials, which prove the association of iron deficiency anemia with *H. pylori* infection and serves as a benchmark for future clinical trials.

## Conclusions

This systematic review focused on whether there was any association between* H. pylori *infection and iron deficiency anemia and the effects of *H. pylori* eradication on iron-related parameters, including serum ferritin, hemoglobin, transferrin saturation, mean corpuscular volume (MCV), and mean corpuscular hemoglobin (MCH) levels. The cross-sectional studies considered for this systematic review revealed a significant association between *H. pylori* infection and low serum ferritin and hemoglobin levels. Randomized clinical trials proved this association by demonstrating improvement in serum ferritin levels and hemoglobin by *H. pylori* eradication treatment. More randomized clinical trials and cohort studies are required in this direction to determine whether *H. pylori* eradication therapy is necessary for patients presenting with iron deficiency anemia which is refractory to treatment with iron therapy alone. The authors hope that this systematic review serves as a template for future studies to actively investigate the gaps in knowledge which we still have in treating refractory iron deficiency anemia and develop guidelines on this issue.
